# Decontaminating N95/FFP2 masks for reuse during the COVID-19 epidemic: a systematic review

**DOI:** 10.1186/s13756-021-00993-w

**Published:** 2021-10-11

**Authors:** Alexandra Peters, Nasim Lotfinejad, Rafael Palomo, Walter Zingg, Pierre Parneix, Hervé Ney, Didier Pittet

**Affiliations:** 1 Infection Control Programme and WHO Collaborating Center on Patient Safety, University of Geneva Hospitals and Faculty of Medicine, 4 Rue Gabrielle-Perret-Gentil, 1211 Geneva 14, Switzerland; 2 University of Geneva, Geneva, Switzerland; 3 Infection Control Programme, University Hospital of Zürich, Zürich, Switzerland; 4 Nouvelle Aquitaine Healthcare-Associated Infection Control Centre, Bordeaux University Hospital, Bordeaux, France

## Abstract

**Background:**

With the current COVID-19 pandemic, many healthcare facilities have been lacking a steady supply of filtering facepiece respirators. To better address this challenge, the decontamination and reuse of these respirators is a strategy that has been studied by an increasing number of institutions during the COVID-19 pandemic.

**Methods:**

We conducted a systematic literature review in PubMed, PubMed Central, Embase, and Google Scholar. Studies were eligible when (electronically or in print) up to 17 June 2020, and published in English, French, German, or Spanish. The primary outcome was reduction of test viruses or test bacteria by log3 for disinfection and log6 for sterilization. Secondary outcome was physical integrity (fit/filtration/degradation) of the respirators after reprocessing. Materials from the grey literature, including an unpublished study were added to the findings.

**Findings:**

Of 938 retrieved studies, 35 studies were included in the analysis with 70 individual tests conducted. 17 methods of decontamination were found, included the use of liquids (detergent, benzalkonium chloride, hypochlorite, or ethanol), gases (hydrogen peroxide, ozone, peracetic acid or ethylene oxide), heat (either moist with or without pressure or dry heat), or ultra violet radiation (UVA and UVGI); either alone or in combination. Ethylene oxide, gaseous hydrogen peroxide (with or without peracetic acid), peracetic acid dry fogging system, microwave-generated moist heat, and steam seem to be the most promising methods on decontamination efficacy, physical integrity and filtration capacity.

**Interpretation:**

A number of methods can be used for N95/FFP2 mask reprocessing in case of shortage, helping to keep healthcare workers and patients safe. However, the selection of disinfection or sterilization methods must take into account local availability and turnover capacity as well as the manufacturer; meaning that some methods work better on specific models from specific manufacturers.

***Systematic registration number*:**

CRD42020193309.

**Supplementary Information:**

The online version contains supplementary material available at 10.1186/s13756-021-00993-w.

## Introduction

With the current COVID-19 pandemic, healthcare facilities have suffered from shortage of N95 or filtering facepiece 2 (FFP2) respirators due to concurrent increased demand and decreased production capacity and supply interruptions, or lack of resources. This emergency situation warrants the taking of extraordinary measures to maintain the security of health workers delivering care to COVID-19 patients. Decontamination and reuse of N95/FFP2 respirators is a promising solution, which has been envisaged by an increasing number of institutions all over the world during the first wave of the COVID-19 pandemic.

N95/FFP2 respirators are single use personal protective equipment and thus, their reprocessing was underexplored. Over the past decades, the idea of reprocessing N95/FFP2 respirators emerged during outbreaks due to SARS-CoV and influenza, but given that these events were geographically or temporally limited compared to the COVID-19 pandemic, there was no urge to proceed to legally challengeable protocols of reprocessing single use devices. The COVID-19 pandemic urged various stakeholders to consider N95/FFP2 respirator reprocessing as an alternative to protect health workers at the frontline. The main challenge today is to find reliable information on safe reprocessing within the vast quantity of publications on COVID-19 in the last months. Although in this review we focus on the efficacy of decontamination methods, it goes without saying that respirators should be reprocessed only if no better options are available. As most of the studies have so far been performed by industry, it is important for reviews on the topic to take the grey literature into account.

In order to have a clear understanding of the research that has already been conducted, we performed a systematic literature review for the question: *What are the tested methods for decontaminating N95/FFP2 respirators, and what is the efficacy of those methods on viral contamination?* The primary outcome was efficacy on reducing pathogens (viruses or bacteria) on pre-contaminated N95/FFP2 respirators. The secondary outcome looked at the physical integrity (fit/filtration/degradation) of the masks after the decontamination process.

The overall aim of this systematic review was to assess the microbiological efficacy of decontamination methods of N95/FFP2 respirators. We hope that this overview of the work performed thus far will help orient further research as well as help healthcare facilities make decisions regarding methods of respirator decontamination.

## Methods

We performed a systematic review protocol according to the PRISMA checklist [1]. Considering that we expected many papers of interest not being available in PubMed, Cochrane or Embase, we broadened our search strategy towards PubMed Central and Google Scholar. The results were contextualized with further material from the grey literature, including unpublished data from the Geneva University Hospitals (HUG) laboratory and partner institutions.

All controlled original studies were eligible when applying a quantitative study design and measuring the effect of a decontamination strategy on a microbiological outcome such as respiratory viruses or bacteria, and published until the 17th of June, 2020. Studies with an English abstract were eligible when published in English, French, German, or Spanish.

We applied to following search strategy: (N95 OR FFP2 OR KN95) AND (decontamination OR disinfection OR sterilization) AND (reuse OR reprocessing OR reusing) AND (coronavirus OR “COVID 19” OR "SARS CoV-2" OR stearothermophilus OR influenza). The full search strategies are available in the Additional file 1. We included stearothermophilus in our search terms because it often used as an indicator pathogen decontamination testing, and influenza because we thought that it would possibly be a virus that would often be tested due to the 2009 pandemic. We included all studies that came up through the search and matched our search criteria, meaning that results from tests on bacteria and bacteriophages were also included in the table.

Results were stratified by the decontamination methods test organisms used, and whether the effect was disinfection (≥ log 3 reduction of the test microorganism) or sterilization (≥ log 6 reduction of the test microorganism). Any decontamination method was eligible if measuring its efficacy on a microorganism compared to a control. Primary outcome was disinfection or sterilization of the test microorganism. Therefore, all studies included were compatible with this outcome domain. Secondary outcomes included physical integrity, fit testing, and filtration capacity after reprocessing, and not all studies had data on this. We excluded articles other than original research.

The “study population” included N95, KN95, or FFP2 respirators or their equivalents. Outcomes were designated as “sterilization”, “disinfection” and “failure”. “Failure” was defined as having a lower than 3 log reduction of the test microorganism.

Titles and abstracts were screened independently by two reviewers. Duplicate articles were removed. After deduplication, 961 articles were screened. The full text of all potentially eligible studies was independently assessed by at least two authors. Disagreements were resolved by consensus or by consulting a third reviewer where necessary. Authors tabulatied the study intervention characteristics and compared them against the planned groups for synthesis. Inclusions and exclusions were recorded following the PRISMA guidelines, and reasons for exclusion were detailed. Data of each study was extracted by two review authors. The standardized extraction forms collected the following information: study title, author; year of publication; intervention(s), microorganism tested, microbiological outcome (log reduction), whether authors recommended their method of decontamination, whether disinfection or sterilization was achieved, data on physical integrity when available (including fit or filtration), and comments. These elements were included in the table (see Table 1), and were organized by type of intervention. These types of (liquid, gasses, heat, UV) were analyzed as individual subgroups.

All included studies were assessed for their inclusion by at least two review authors. All eligible studies were in-vitro studies; thus, only studies testing a method against a control were eligible. Studies that were finally not included were usually because the outcome didn’t quantify a microbiological reduction. We expected to identify a large variety of procedures and methodologies. Thus, a descriptive analysis with narrative synthesis was planned. Ethical approval was not required for this review.

## Results

**Fig. 1:**
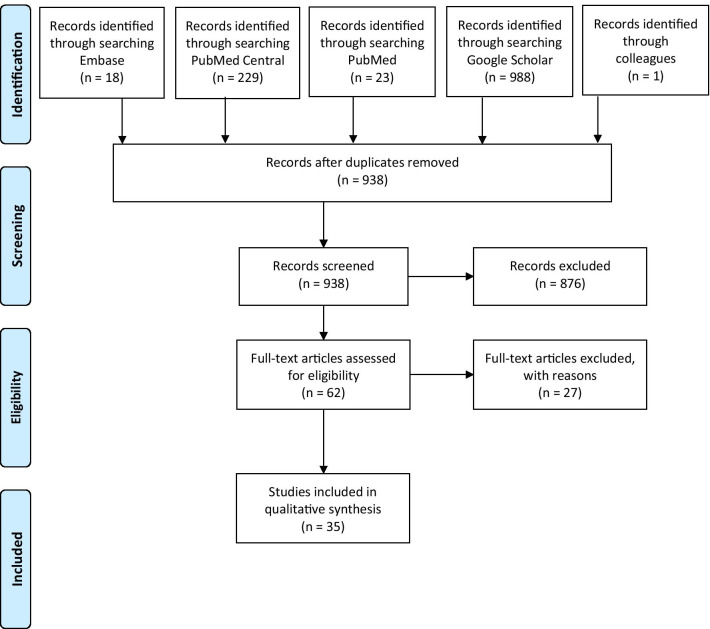
Decontaminating N95/FFP2 masks for reuse during the COVID-19 epidemic; study Prisma flow chart

Our search identified a total of 23 publications from PubMed, 18 from Embase, 0 from Cochrane, 229 from Pubmed Central, and 988 from Google Scholar. Originally, there were 1010 results using Google Scholar, but Google Scholar only allows access to the first 99 pages of results; and thus, we were not able to assess the last 22 articles from the Google Scholar results (Fig. 1). A total of 35 studies were included for final analysis [2–36]. The following decontamination methods were identified (Table 1 and see complete table in the on-line Appendix): (saturated) steam (with pressure; autoclave), moist heat (without pressure; devices other than autoclaves), dry heat, UVGI (UVC and UVA), hydrogen peroxide vapor (HPV) and its associated forms (aerosolized or ionized) either alone or in combination with peracetic acid, peracetic acid (dry fogging system), ethylene oxide, ozone, ethanol, sodium hypochlorite, benzalkonium chloride, and detergent (non-antimicrobial) wipes. Individual studies were not assessed for bias or for risk of reporting bias. As a number of these small, in vitro studies were conducted my industry, it is possible that unfavorable results were not published as often.

**Table 1:** Decontaminating N95/FFP2 masks for reuse: results of the systematic review 2011–June 2020

Title	Authors	Intervention	Microorganisms tested	Quantification method	Outcome: log reduction compared to control	Data on physical integrity/fit/filtration/residue	Sterilization/probable disinfection (minimum 3-log reduction for disinfection, 6-log for sterilization)	Method recommended (see text for the definition of this column)	Comments
*Liquids*									
Cleaning of filtering facepiece respirators contaminated with mucin and Staphylococcus aureus [2]	Heimbuch et al	Benzalkonium chloride (Wipes)	Mucin or Staphylococcus aureus	CFU assay	3–5	Fail	Disinfection	No	
Relative survival of Bacillus subtilis spores loaded on filtering facepiece respirators after five decontamination methods [3]	Lin et al	Ethanol 50–95%	Bacillus subtilis spores	CFU assay	N/A, but culture results were positive	N/A	Failed	No	
Effect of various decontamination procedures on disposable N95 mask integrity and SARS-CoV-2 infectivity [4]	Smith et al	Ethanol 70%	SARS-CoV-2	RT-PCR	likely > 3 log	Fail	Disinfection	No	The initial contamination was at 3 log as it was coming from human sample
Effectiveness of N95 Respirator Decontamination and Reuse against SARS-CoV-2 Virus [5]	Fischer et al	Ethanol 70% for 10 min	SARS-CoV-2 (HCoV-19 nCoV-WA1-2020 (MN985325.1))	TCID50	4	Fail	Disinfection	No	
Relative survival of Bacillus subtilis spores loaded on filtering facepiece respirators after five decontamination methods [3]	Lin et al	Hypochlorite in a 0.54% solution	Bacillus subtilis spores	CFU assay	N/A, but culture results were negative	N/A	Disinfection	N/A	No colonies but no info on log reduction
Cleaning of filtering facepiece respirators contaminated with mucin and Staphylococcus aureus [2]	Heimbuch et al	Hypochlorite (Wipes) in a 0.9% solution	S. aureus	CFU assay	4–5 except for nose pads	Fail	Disinfection and failure	No	All masks had good disinfection except for on the nose pads (less than 2 log reduction)
Cleaning of filtering facepiece respirators contaminated with mucin and Staphylococcus aureus [2]	Heimbuch et al	Nonantimicrobial detergent wipes	Mucin or Staphylococcus aureus	CFU assay	1	Fail	Failure	No	
*Heat*									
Effectiveness of N95 Respirator Decontamination and Reuse against SARS-CoV-2 Virus [5]	Fischer et al	Dry heat at 70 °C for 10–60 min	SARS-CoV-2 (HcoV-19 nCoV-WA1-2020 (MN985325.1)	TCID50	> 1to > 3 depending on time	Pass (max 3 rounds)	Disinfection and failure	Yes	Ability to disinfect was time dependent
Effectiveness of Ultraviolet-C Light and a High-Level Disinfection Cabinet for Decontamination of N95 Respirators [6]	Cadnum et al	Dry heat at 70 °C for 30 min	Staphylococcus aureus (MRSA) and bacteriophages MS2 and Phi6	CFU assay, Plaque assay	Bacteriophages < 1, MRSA > 4	N/A	Disinfection and failure	No	Failure for bacteriophages
It’s not the heat, it’s the humidity: Effectiveness of a rice cooker-steamer for decontamination of cloth and surgical face masks and N95 respirators [7]	Li et al	Dry heat at 100 °C for 15 min	MS2 ad MRSA	Plaque assay	< 3 log10 reduction	Pass (visual)	Failure	No	
Validation of N95 filtering facepiece respirator decontamination methods available at a large university hospital [8]	Wigginton et al	Dry heat at 82 °C for 30 min	Staphylococcus aureus and Geobacillus stearothermophilus	Plaque assay	S. aureus: < 1.0 log10, G. stearotherophilus: < 0.3 log10	Pass	Failure	No	
Decontamination of face masks and filtering facepiece respirators via ultraviolet germicidal irradiation, hydrogen peroxide vaporization, and use of dry heat inactivates an infectious SARS-CoV-2 surrogate virus [9]	Ludwig-Begall et al	Dry heat at 102 °C for 60 min	Porcine respiratory coronavirus (PRCV)	TCID50	≥ 4	N/A	Disinfection	N/A	
Decontamination of Surgical Face Masks and N95 Respirators by Dry Heat Pasteurization for One Hour at 70°C [10]	Xiang et al	Dry heat at 60 °C and 70 °C 60 min	E. coli, S. aureus, P. aeruginosa, K. pneumonia, A.baumannii, C. pseudodiphtheria, and C.albicans, Inf A virus (H1N1)	TCID 50	N/A, but culture results were negative	Pass	Disinfection	Yes	
Dry Heat as a Decontamination Method for N95 Face Respirator Reuse [11]	Oh et al	Dry heat at 120 °C for 50 min	Tulane virus, rotavirus, adenovirus, transmissible gastroenteritis virus	Plaque assay	Tulane > 5.2, rotavirus > 6.6, adenovirus > 4.0, gastroenteritis > 4.7	Pass	Sterilization and disinfection	Yes	All disinfection except for rotavirus which reached sterilization
Thermal Disinfection Inactivates SARS-CoV-2 in N95 Respirators while Maintaining Their Protective Function [12]	Daeschler et al	Dry heat at 70 °C for 60 min	SARS-CoV-2 and E. coli	TCID 50	mixed: > 4 for SARS CoV-2, < 1 for E.coli	Pass	Disinfection and failure	Depends	Failure for E.Coli. Recommendation dependent on microorganism present
Relative survival of Bacillus subtilis spores loaded on filtering facepiece respirators after five decontamination method [3]	Lin et al	Dry Heat (electric rice cooker) 149–164 °C	Bacillus subtilis spores	CFU assay	N/A, culture results were mixed	N/A	Failure	N/A	Possibly disinfection after 24 h, but not immediately
Efficacy of moist heat decontamination against various pathogens for the reuse of N95 respirators in the COVID-19 emergency [13]	Oral et al	Moist heat at 60 °C at 80% humidity for 30 min	Bovine viral diarrhea virus (BVDV), Porcine Parvovirus (PPV) and Influenza A Virus, S. aureus, P. Aeruginosa and A. Baumanii	cell culture	S. aureus: 5.32 P.aeruginosa:5.7 A. Baumannii:5.92 InfA:4.35 modestly BVDV:1.62 PPV:0	N/A	Disinfection and failure	N/A	Failure for BVDV and PPV
A pandemic influenza preparedness study: Use of energetic methods to decontaminate filtering facepiece respirators contaminated with H1N1 aerosols and droplets [14]	Heimbuch et al	Moist heat at 65 °C at 85% humidity for 30 min	H1N1	TCID50	> 4	Pass	Disinfection	Yes	There was only a visual examination for fit and integrity
Biological Aerosol Test Method and Personal Protective Equipment (PPE) Decon [15]	Hinrichs et al	Moist heat at 62 °C at 85% humidity for 20 min	influenza virus A (H5N1)	RT-PCR and TCID50	≥ 4	N/A	Disinfection	N/A	
Effect of moist heat decontamination on methicillin-sensitive S. aureus for the reuse of N95 respirators in the COVID-19 emergency [16]	Gil et al	Moist heat at 60 °C at 80% humidity for 30 min	S. aureus (methicillin sensitive)	CFU assay	5.31	N/A	Disinfection	N/A	
Thermal Disinfection Inactivates SARS-CoV-2 in N95 Respirators while Maintaining Their Protective Function [12]	Daeschler et al	Moist heat at 70 °C at 50% humidity for 60 min	SARS-CoV-2 and E. coli	TCID 50	mixed: > 4 for SARS CoV-2, < 3 for E.coli	Pass	Disinfection and failure	Depends	Failure for E.Coli because initial contamination was too low, but was probably disinfection. Recommendation dependent on microorganism present
Effectiveness of three decontamination treatments against influenza virus applied to filtering facepiece respirators [17]	Lore et al	Moist heat at 65 °C for 20 min	H5N1	TCID50	≥ 4.62 and ≥ 4.65	Pass	Disinfection	Yes	
It’s not the heat, it’s the humidity: Effectiveness of a rice cooker-steamer for decontamination of cloth and surgical face masks and N95 respirators [7]	Li et al	Moist heat for 12–15 min	MS2 ad MRSA	Plaque assay	> 5 log10 reduction	Pass	Disinfection	Yes	There was only a visual examination for fit and integrity
Validation of N95 filtering facepiece respirator decontamination methods available at a large university hospital [8]	Wigginton et al	Moist heat at 80 °C at 60% humidity for 30 min	MS2, phi6, influenza A virus S aureus, G. stearotherophilus	Plaque assay	MS2: > 6.8, Phi6: > 6.6, influenza virus: > 3.4, and MHV > 1.4, S disinfection > 2.9, G. stearotherophilus < 0.3	Pass	Sterilization, disinfection and failure	No	MS2: Sterilization, Phi6: Sterilization, Influenza virus: Disinfection, MHV: failed, S aureus: failed, G. stearotherophilus: failed
A pandemic influenza preparedness study: Use of energetic methods to decontaminate filtering facepiece respirators contaminated with H1N1 aerosols and droplets [14]	Heimbuch et al	Moist heat (microwave-generated) for 2 min	H1N1	TCID50	> 4	Pass	Disinfection	Yes	There was only a visual examination for fit and integrity
Biological Aerosol Test Method and Personal Protective Equipment (PPE) Decon [15]	Hinrichs et al	Moist heat (microwave-generated) for 2 min	influenza virus AH5N1	RT-PCR and TCID50	≥ 4	N/A	Disinfection	N/A	
Effectiveness of three decontamination treatments against influenza virus applied to filtering facepiece respirators [17]	Lore et al	Moist heat (microwave-generated) for 2 min	H5N1	TCID50	≥ 4.81 and ≥ 4.79	Pass	Disinfection	Yes	
Evaluation of microwave steam bags for the decontamination of filtering facepiece respirators [18]	Fisher et al	Moist heat (microwave-generated) for 1.5 min	MS2 bacteriophage	CFU assay	3.10 – 4.64	Mixed	Disinfection	Mixed	Can be only recommended in some cases, depending on model of the mask and how much water is absorbed. Some failure for physical integrity/fit/filtration
Microwave-Generated Steam Decontamination of N95 Respirators Utilizing Universally Accessible Materials [19]	Zulauf et al	Moist heat (microwave-generated) for 3 min for 1, 5, or 20 cycles	Escherichia coli MS2 bacteriophage	Plaque assay	5–6	Pass	Sterilization and disinfection	Yes	Average 6-log10 PFU and a minimum 5-log10 PFU reduction after a single three-minute microwave treatment
Steam treatment for rapid decontamination of N95 respirators and medical face masks [20]	Li et al	Steam (autoclave, short cycle) at 100 °C for 10–30 s	S. aureus (MRSA), G. stearothermophilus spores, bacteriophage MS2	CFU assay	MS2 and MRSA > 3, G. stearothermophilus spores: fail	Pass	Disinfection and failure	No	Failure for G. stearothermophilus spores. Authors also tested a 2 s cycle, but the test failed
Relative survival of Bacillus subtilis spores loaded on filtering facepiece respirators after five decontamination methods [3]	Lin et al	Steam (autoclave) at 121 °C for 15 min	Bacillus subtilis spores	CFU assay	N/A, but culture results were negative	N/A	Disinfection	N/A	No colonies but no info on log reduction
N95 mask decontamination using standard hospital sterilization technologies [21]	Kumar et al	Steam (autoclave) at 121 °C for 40 min	Vesicular stomatitis virus, Indiana serotype (VSV) or SARSCoV-2 (contaminated group)	TCID50	VSV: > 6, SARSCoV-2: 5.2–6.3	Pass	Disinfection and Sterilization	Yes	Some FFRs may have had too low a level of contamination to ensure a 6-log reduction
*Gases*									
N95 mask decontamination using standard hospital sterilization technologies [21]	Kumar et al	Ethylene oxide (EtO) for 60 min	Vesicular stomatitis virus, Indiana serotype (VSV)	TCID50	VSV: > 6	Pass	Sterilization	No	
Validation of N95 filtering facepiece respirator decontamination methods available at a large university hospital [8]	Wigginton et al	Ethylene oxide (EtO) 55 °C for 60 min at 45% RH	MS2	Plaque assay	> 5.8	Pass	Disinfection	No	
Validation of N95 filtering facepiece respirator decontamination methods available at a large university hospital [8]	Wigginton et al	Hydrogen peroxide (gaseous HPGP) in a 59% solution for 24 min	MS2, phi6, influenza A virus	Plaque assay	Phi6: > 7.9, influenza virus > 3.8, MS2: 5.6	Pass	Sterilization and disinfection	No	Sterilization for Phi6, disinfection for influenza virus and MS2
Effect of various decontamination procedures on disposable N95 mask integrity and SARS-CoV-2 infectivity [4]	Smith et al	Hydrogen peroxide (gaseous HPV) in a 30% solution (500 ppm) at humidity between 38–99.5% for 20 min	SARS-CoV-2	RT-PCR	2 masks: ~ five log10 reduction < 3 log	Pass	Disinfection	No	The initial contamination was at 3 log as it was coming from human sample
Aerosolized Hydrogen Peroxide Decontamination of N95 Respirators, with Fit-Testing and Virologic Confirmation of Suitability for Re-Use During the COVID-19 Pandemic [22]	Derr et al	Hydrogen Peroxide (Gaseous-aHP) in a 7% solution for 12 min	SARS-CoV-2, Herpes simplex virus 1, Coxsackie virus B3, Pseudomonas phi6 bacteriophage	Plaque assay	N/A, but culture results were negative	Pass	Sterilization	Yes	
Vapor H2O2 sterilization as a decontamination method for the reuse of N95 respirators in the COVID-19 emergency [23]	Oral et al	Hydrogen Peroxide (Gaseous-HPV) 410 ppm for 180 min	SARS-CoV-2	Plaque assay	> 2.6	Pass	Disinfection	Yes	The initial contamination was too low to be able to detect sterilization
N95 mask decontamination using standard hospital sterilization technologies [21]	Kumar et al	Hydrogen peroxide (gaseous-HPV) in a 35% solution (750 ppm) for 60 min	Vesicular stomatitis virus, Indiana serotype (VSV) or SARSCoV-2 (contaminated group)	TCID50	VSV: > 6, SARSCoV-2: 5.2–6.3	Pass	Sterilization and disinfection	Yes	Some FFRs may have had too low a level of contamination to ensure a 6-log reduction
Effectiveness of N95 Respirator Decontamination and Reuse against SARS-CoV-2 Virus [5]	Fischer et al	Hydrogen peroxide (gaseous-HPV) (1000 ppm) for 10 min	SARS-CoV-2 (HCoV-19 nCoV-WA1-2020 (MN985325.1))	TCID50	> 4	Pass	Disinfection	Yes	
Validation of N95 filtering facepiece respirator decontamination methods available at a large university hospital [8]	Wigginton et al	Hydrogen peroxide (gaseous-HPV) 446–659 ppm	MS2, phi6, influenza A virus, murine hepatitis virus, E. coli, S. aureus, G. stearothermophilus, A. niger	Plaque assay	> 2	Pass	Failure	No	
Decontamination of face masks and filtering facepiece respirators via ultraviolet germicidal irradiation, hydrogen peroxide vaporization, and use of dry heat inactivates an infectious SARS-CoV-2 surrogate virus [9]	Ludwig-Begall et al	Hydrogen peroxide (gaseous-HPV) in a 59% solution (750 ppm) for 28 min	Porcine respiratory 38 coronavirus (PRCV)	TCID50	≥ 5	N/A	Disinfection	N/A	
Hydrogen Peroxide Vapor sterilization of N95 respirators for reuse [24]	Kenney et al	Hydrogen peroxide (gaseous-HPV) 30–40-min gassing phase at 16 g/min	Phages phi-6, T7 and T1	Plaque assay, TCID50	N/A, but complete eradication of phages from masks	Pass	Sterilization	Yes	Limit of detection was 5PFU, lower than infectious dose, and authors used the term "sterilization"
Disinfection of N95 respirators by ionized hydrogen peroxide during pandemic coronavirus disease 2019 (COVID-19) due to SARS-CoV-2 [25]	Cheng et al	Hydrogen Peroxide (Gaseous-iHP) in a 7.8% solution	Influenza A virus subtype H1N1	TCID 50	N/A, but culture results were negative	N/A	Disinfection	N/A	No growth, but no specific log reduction mentioned, paper uses term "disinfection"
N95 mask decontamination using standard hospital sterilization technologies [21]	Kumar et al	Hydrogen peroxide (gaseous-LT-HPGP) in a 59% solution for 47 min	Vesicular stomatitis virus, Indiana serotype (VSV)	TCID50	VSV: > 6	Pass	Sterilization	No	LT-HPGT-treated masks failed testing beyond the first cycle
Effectiveness of Ultraviolet-C Light and a High-Level Disinfection Cabinet for Decontamination of N95 Respirators [6]	Cadnum et al	Hydrogen Peroxide (Gaseous) and Peracetic acid for 1–3 cycles of 21 min, and a single cycle of 31 min	S. aureus (MRSA) and bacteriophages MS2 and Phi6	CFU assay, Plaque assay	1 cycle: > 2.1, 2 cycles: > 3.6, 3 cycles > 6 log10	N/A	Sterilization, disinfection, failure	N/A	Outcome was dependent on the number of cycles (3 cycles resulted in sterilization)
Scalable In-hospital Decontamination of N95 Filtering Facepiece Respirator with a Peracetic Acid Room Disinfection System [26]	John et al	Hydrogen Peroxide (Gaseous) and Peracetic acid in a 18% solution at at 20 °C for 12–19 min	MS2 bacteriophage and G. stearothermophilus spores	CFU assay	6/6/4	Pass	Sterilization and disinfection	Yes	Shorter cycle led to disinfection. Can't be used with masks containing cellulose
Enveloped Virus Inactivation on Personal Protective Equipment by Exposure to Ozone [27]	Blanchard et al	Ozone at 20 ppm and 70% humidity for 40 min	Influenza virus A A/WSN/33, RSV A2	Plaque assay	4	Mixed	Disinfection	Mixed	Although the facepiece was unaffected for fit/filtration, the elastic band failed
Fast and easy disinfection of coronavirus-contaminated face masks using ozone gas produced by a dielectric barrier discharge plasma generator [28]	Lee et al	Ozone at 120 ppm for 1 and 5 min	HCoV-229E	TCID 50	3	Pass	Disinfection	Yes	The initial contamination was too low to be able to detect sterilization
Disinfection of N95 Respirators with Ozone [29]	Manning et al	Ozone at 450 ppm and 75–90% humidity for 120 min	Pseudomonas aeruginosa	CFU assay	> 7–> 9, (one sample failed disinfection-1.38 log reduction)	Mixed	Sterilization and failure	Mixed	There was a single failure, but one needs to verify why one mask failed the test. Although the facepiece was unaffected for fit/filtration, the elastic band failed
N95 mask decontamination using standard hospital sterilization technologies [21]	Kumar et al	Peracetic acid dry fogging system (PAF) at 80–90% humidity for 60 min	Vesicular stomatitis virus, Indiana serotype (VSV) or SARSCoV-2 (contaminated group)	TCID50	VSV: > 6, SARSCoV-2: 5.2–6.3	Pass	Disinfection and sterilization	Yes	Some FFRs may have had too low a level of contamination to ensure a 6 log reduction
*Ultra violet light*									
Relative survival of Bacillus subtilis spores loaded on filtering facepiece respirators after five decontamination methods [3]	Lin et al	UVA at 365 nm and 1.87–37.44 J/cm2 for 1–20 min	Bacillus subtilis spores	CFU assay	N/A, but culture results were positive	N/A	Failed	No	
A Scalable Method for Ultraviolet C Disinfection of Surgical Facemasks Type IIR and Filtering Facepiece Particle Respirators 1 and 2 [30]	Lede et al	UVGI at 253.7 nm and 6 lamps, each 0.6 J/cm2 for 40 min	S. aureus	CFU assay	7	Pass	Sterilization	Yes	
Ultraviolet germicidal irradiation of influenza-contaminated N95 filtering facepiece respirators [31]	Mills et al	UVGI at 254 nm and 1.1 J/cm2 and 48% humidity for 40 min	Influenza virus (H1N1)	TCID 50	≥ 3 log on 12 of 15 FFR models and straps from 7 of 15 FFR models	N/A	Disinfection and failure	N/A	
A method to determine the available UV-C dose for the decontamination of filtering facepiece respirators [32]	Fisher and Shaffer	UVGI at 254 nm and 0.15–1.5 J/cm2 for 1–10 min	MS2 coliphage	Plaque assay	minimum IFM dose of 1000 J m^-2: log reduction > = 3	Mixed	disinfection	No	Model dependent outcomes. Model-specific exposure times to achieve this IFM dose Ranged from 2 to 266 min. Mostly failure for physical integrity/fit/filtration
The Effect of Ultraviolet C Radiation Against SARS-CoV-2 Inoculated N95 Respirators [33]	Ozog et al	UVGI at 254 nm and 1.5 J/cm2 for 60–70 s/side	SARS-CoV-2	TCID 50	N/A, culture results were mixed	N/A	Disinfection and failure	N/A	Disinfection but not for all models of masks (5 models of N95 tested)
Effects of relative humidity and spraying medium on UV decontamination of filters loaded with viral aerosols [34]	Woo et al	UVGI at 254 nm and 1.8 and 3.6 J/cm2 and 30,60, and 90% humidity for 30 and 60 min	MS2	Plaque assay	mixed, highest inactivation efficiency: 5.8 log	N/A	Disinfection and failure	N/A	Disinfection, but not for all masks and conditions
Relative survival of Bacillus subtilis spores loaded on filtering facepiece respirators after five decontamination methods [3]	Lin et al	UVGI at 254 nm and 1.13–22.68 J/cm2 for 1–20 min	Bacillus subtilis spores	CFU assay	N/A, culture results were mixed	N/A	Failure	N/A	Possible disinfection after 24 h, but not immediately
Effectiveness of three decontamination treatments against influenza virus applied to filtering facepiece respirators [17]	Lore et al	UVGI at 254 nm and 18 kJ/m2 for 15 min	H5N1	TCID50	≥ 4.54 and ≥ 4.65	Pass	Disinfection	Yes	
A pandemic influenza preparedness study: Use of energetic methods to decontaminate filtering facepiece respirators contaminated with H1N1 aerosols and droplets [14]	Heimbuch et al	UVGI at 254 nm and 18 kJ/m2 for 15 min CHECK SAME AS LORE)	H1N1	TCID50	> 4	Pass	Disinfection	Yes	There was only a visual examination for fit and integrity
Effectiveness of N95 Respirator Decontamination and Reuse against SARS-CoV-2 Virus [5]	Fischer et al	UVGI at 260-285 nm and 0.33 J/cm2, 0.99 J/cm2, and 1.98 J/cm2 for 10, 30, and 60 min	SARS-CoV-2 (HCoV-19 nCoV-WA1-2020 (MN985325.1))	TCID50	between 1 and 3, depending on the time	Pass	Disinfection and failure	Yes	Time-dependent: failure for masks below 60 min, probable disinfection at 60 min
Effectiveness of Ultraviolet-C Light and a High-Level Disinfection Cabinet for Decontamination of N95 Respirators [6]	Cadnum et al	UVGI for 1 and 30 min	S. aureus (MRSA) and bacteriophages MS2 and Phi6	CFU assay, Plaque assay	0–4	N/A	Disinfection and failure	No	Outcome depended on model of mask and pathogen, only 1 of 9 masks qualified as disinfected
Validation of N95 filtering facepiece respirator decontamination methods available at a large university hospital [8]	Wigginton et al	UVGI at 200-315 nm for 5 min	MS2, phi6, influenza A virus, murine hepatitis virus, E. coli, S. aureus, G. stearothermophilus, A. niger	Plaque assay	MS2: 0.7 – 1.3, Phi6: 0.2 – 1.8, influenza: 1.4 – 1.7, MHV > 1.4, S. aureus < 1.0, G. stearotherophilus < 0.3 log10	Pass	Failure	No	
Effect of various decontamination procedures on disposable N95 mask integrity and SARS-CoV-2 infectivity [4]	Smith et al	UVGI at 254 nm and 0.63 J/cm2 for 33 min	SARS-CoV-2	RT-PCR	< 3 log	Fail	Neither	No	The initial contamination was at 3 log as it was coming from human sample
Decontamination of face masks and filtering facepiece respirators via ultraviolet germicidal irradiation, hydrogen peroxide vaporisation, and use of dry heat inactivates an infectious SARS-CoV-2 surrogate virus [9]	Ludwig-Begall et al	UVGI at 254 nm and 5.2 J/cm [2] for 4 min	Porcine respiratory 38 coronavirus (PRCV)	TCID50	≥ 4	N/A	Disinfection	N/A	
Disinfection effect of pulsed xenon ultraviolet irradiation on SARS-CoV-2 and implications for environmental risk of COVID-19 transmission [35]	Simmons et al	UVGI for 5 min	SARS CoV-2	Plaque assay	> 4.79	N/A	Disinfection	N/A	
Biological Aerosol Test Method and Personal Protective Equipment (PPE) Decon [15]	Hinrichs et al	UVGI at 254 nm and 18 kJ/m2 for 15 min	influenza virus AH5N1	RT-PCR and TCID50	≥ 4 log10 TCID50	N/A	Disinfection	N/A	
Reusability of filtering facepiece respirators after germicidal UV irradiation [36]	Vernez et al	UVGI + dry heat (Dry Heat at 70 °C for 15 min and then UVGI at 254 nm and 60 mJ/cm2 for 4 min)	vB_HSa_2002 and P66 phages	Plaque assay	> 3	Pass	Disinfection	Yes	
Validation of N95 filtering facepiece respirator decontamination methods available at a large university hospital [8]	Wigginton et al	UVGI + dry heat (Dry heat at 82 °C and UVGI at 200–315 nm)	MS2, phi6, influenza A virus, murine hepatitis virus, Staphylococcus aureus	Plaque assay	The influenza virus: > 3.9, the mouse coronavirus: 1.1, Phi6 deposited in PBS < 1.5 when heated to 82C and at ~ 8% RH. S. aureus: 1.2	Pass	Disinfection and failure	No	Influenza virus: Disinfection MHV: Failed MS2: Failed, Phi6: Failed S. aureus: failed
Validation of N95 filtering facepiece respirator decontamination methods available at a large university hospital [8]	Wigginton et al	UVGI + medium humidity heat (Heat at 80 °C, RH at 62–66% and UVGI at 200–315 nm for 15 min)	MS2, phi6, influenza A virus	Plaque assay	influenza virus: > 3.9, mouse coronavirus MHV > 1.1, MS2 > 6.8, Phi6 > 6.6	Pass	Sterilization, disinfection, failure	No	MS2: Sterilization, Phi6: Sterilization, Influenza virus: Disinfection, MHV: failed

Procedures of the methods were generally well described. Nine studies were performed before 2020, with the oldest publication from 2011. Many of these were performed in the context of past influenza pandemics (4 of the 9 studies used a strain of influenza as a test organism). In total, 70 individual tests were conducted, with 14 of the 35 studies evaluating more than one method. Sample sizes ranged from 3 to 115 tested respirators. Often, information regarding the sample size was unclear. The overwhelming majority of the experiments applied heat at different humidity levels, UVGI, or gaseous hydrogen peroxide (Table 1). Details regarding the studies such as the type of intervention, microorganisms tested, quantification methods, log reduction compared to control, data on physical integrity, fit, and filtration, and whether the method is recommended are provided in the table).

Numerous chemicals and processes can be used for disinfecting or sterilizing unsoiled N95/FFP2 respirators. None of these methods are new [37], but recognized processes for disinfecting or sterilizing medical equipment. Overall, 17 distinct processes (or distinct combinations of processes) were tested (see Table 2).

**Table 2:** Number of individual experiments per method of decontamination

Method	Number of studies
Benzalkonium Chloride wipes [2]	1
Non-antimicrobial detergent wipes [2]	1
UVGI + medium humidity heat [8]	1
Peracetic acid dry fogging system [21]	1
UVA [3]	1
Ethylene oxide [8, 21]	2
Hypochlorite [2, 3]	2
UVGI + dry heat [8, 36]	2
Gaseous hydrogen peroxide with peracetic acid [6, 26]	2
Ozone [27–29]	3
Ethanol [3–5]	3
Steam [3, 20, 21]	3
Microwave-generated moist heat [14, 15, 17–19]	5
Moist heat [7, 8, 12–17]	8
Dry Heat [3, 5–12]	9
Gaseous hydrogen peroxide [4, 5, 8, 9, 21–25]	11
UVGI [3–6, 8, 9, 30–35]	15

## Discussion

### Prior knowledge and what this manuscript contributes

With the current COVID-19 pandemic, many healthcare facilities have been lacking a steady supply of filtering facepiece respirators (N95/FFP2). To better address this challenge, the decontamination and reuse of these respirators is a strategy that has been studied by an increasing number of institutions during the COVID-19 pandemic. Although the initial shortages in high-income countries have been resolved for the moment, this area of study is still crucial for low-resource areas, as we head into yet another wave of COVID-19. Learning how to navigate shortages of N95 masks safely, will no doubt have a positive impact on healthcare delivery in the future.

Prior to COVID-19, there were very few studies that looked into the reprocessing of respirators. One looked at S. aureus and mucin contamination [2], one looked at Bacillus subtilis spores, 3 three on MS2 bacteriophages [18, 32, 34], and four on influenza viruses [14, 15, 17, 31]. A review on the same subject was published by Rodriguez-Martinez et al. as a preprint in July 2020 while we were still working on our review [38]. Still, our review is more comprehensive, as we took all of the grey literature into account, where the majority of the studies were published. Therefore, this review has over twice the number of studies included. This means that it provides far more resources for healthcare facilities, especially in low resource settings, that still do not have access to the supplies they need today.

### Log reductions

The accepted log reductions for disinfection and sterilization and 4–5 log (depending on whether bacteria/yeasts/or viruses) and 6-log, respectively. However, the US food and Drug Administration announced that for reprocessing masks during COVID-19, “bioburden reduction treatment should result in ≥ 3-log (1000-fold) reduction in microbial numbers and is consistent with a Tier 3 bioburden reduction system” [39]. Additionally, log reductions were often shown as a range or a minimum. Studies shows the reduction from 3-5logs, others 4–5 or as greater than 3. Numerous studies using masks contaminated by human samples (where the contamination was at 3log itself) in an effort to replicate real life conditions were unable to show even a 3log reduction, or in some cases it was enough to show disinfection but not sterilization [4, 12, 21, 23, 28]. Sometimes papers didn’t detail log reductions but used the terms disinfection and sterilization, when there was no bacterial growth on the medium [24, 25]. Therefore, due to the danger of excluding methods that worked because the tests weren’t designed to show as big of a log reduction as possible we decided that it would be better to count anything over a 3-log reduction as “probable disinfection”, and use the generally accepted 6-log reduction for sterilization.

Referring to results in general, ethylene oxide, gaseous hydrogen peroxide, gaseous hydrogen peroxide with peracetic acid, peracetic acid dry fogging system, microwave-generated moist heat, and steam seem to be the most promising methods on decontamination efficacy, physical integrity and filtration capacity. However, successful decontamination or failure is not inextricably linked to the physicochemical process. UVGI, for instance, showed both very good results and failures. Thus, technical implementation and engineering may play an equally important role than the physicochemical properties of a method.

Ensuring the safety of these decontamination methods is paramount. Reprocessing procedures using chemicals such EtOH as may leave a concentration that could be harmful for the user due to residual amounts of reprocessing substances or by-products produced during the process. Steps to ensure the safety of such reprocessing methods on porous masks must therefore be taken into consideration and implemented. If masks are reprocessed without determining the residual quantities of chemicals, this could prove harmful to the users.

We conducted a sub-analysis of the results by decontamination method.

*For the decontamination methods using liquids* One experiment tested benzalkonium chloride wipes on bacteria resulting in disinfection; fit and filtration testing failed [2]. Two experiments tested hypochlorite on bacteria (including spore-forming bacteria) resulting in disinfection [2, 3]. Fit and filtration testing was performed in one of the studies and failed. One experiment tested non-antimicrobial detergent wipes on bacteria resulting in a failure to disinfect; fit and filtration testing failed as well [2]. Three experiments tested ethanol on viruses and spore-forming bacteria resulting in a mixture of disinfection and failure; fit and filtration testing failed [3–5].

*For the decontamination methods using gases* Though peracetic acid dry fogging system (PAF) is technically not a gas, dry fogging is a method that functions in a similar context as gas disinfection would, where the mask is enveloped in very small particles of the substance versus being doused in a liquid. Due to this, we have included PAF in this section. One experiment tested PAF on viruses resulting in a mixture of sterilization and disinfection; fit and filtration testing passed [21]. Two experiments tested ethylene oxide on viruses resulting in a mixture of sterilization and disinfection [8, 21]. Fit and filtration testing was performed in both the studies and both passed [8, 21]. Eleven experiments tested gaseous hydrogen peroxide on viruses, bacteria, spore-forming bacteria, and fungus, resulting in a mixture of sterilization, disinfection, and one failure [4, 5, 8, 9, 21–25]. Fit and filtration testing was performed in nine of the studies, and all passed [4, 5, 8, 21–24]. Three experiments tested ozone on viruses and bacteria resulting in a mixture of sterilization, disinfection, and one failure [27–29]. Fit and filtration testing was performed in all of the studies; one passed [28], and the other two passed in the face piece but failed in the elastic band [27, 29].

*For the decontamination methods using heat* Nine experiments tested dry heat on viruses and bacteria resulting in a mixture of disinfection and failure [3, 5–12]; fit and filtration testing was performed on six studies and all passed [5, 7, 8, 10–12]. Three experiments tested steam on viruses, bacteria and spore-forming bacteria, resulting in a mixture of sterilization, disinfection, failure [3, 20, 21]. Fit and filtration testing was performed in two of the studies and both passed [20, 21]. Eight experiments tested moist heat on viruses and bacteria (including spore-forming bacteria), resulting in a mixture of sterilization, disinfection and failure [7, 8, 12–17]. Fit and filtration testing was performed on five studies, and all passed [7, 8, 12, 14, 17]. Five experiments tested microwave-generated moist heat on viruses and bacteria, resulting in disinfection [14, 15, 17–19]. Fit and filtration testing was performed on four studies [14, 17–19]; two passed [14, 17], and one had mixed results [18].

*For the decontamination methods using UV light* Fifteen experiments [3–6, 8, 9, 14, 15, 17, 30–35] tested UVGI on viruses, bacteria (including spore-forming bacteria, and fungus, resulting in a mixture of sterilization, disinfection, and failure. Fit and filtration testing was performed in seven studies [4, 5, 8, 14, 17, 30, 32]; one failed [4], one had mixed results [32], and five passed [5, 8, 14, 17, 30]. One experiment tested UVA on spore-forming bacteria resulting in in a failure to disinfect; fit and filtration testing was not performed [3].

*For the decontamination using combinations of methods* Two experiments tested gaseous hydrogen peroxide with peracetic acid on viruses and bacteria (including spore-forming bacteria) resulting in sterilization [6, 26]; fit and filtration testing was performed for one study [26], which passed. Two experiments [8, 36] tested UVGI with dry heat on viruses and bacteria resulting in a mixture of disinfection and failure; fit and filtration testing passed. One experiment tested UVGI with medium-humidity heat on viruses, resulting in a mixture of sterilization, disinfection, failure; fit and filtration testing passed [8].

The literature around reprocessing N95/FFP2 respirators has grown exponentially in the wake of the current COVID-19 pandemic. A large variety of methods were tested on different masks, and using different procedures. All methods were performed in controlled settings and without taking into account physical soiling by biological material from wearing the masks in daily routine. A number of reports did reflect on the potential risk of pathogens other than SARS-CoV-2 in biological fluids and how, as a consequence, reprocessing would need to be individualized. This means that the encouraging results from a number of these in vitro studies may not be applicable to healthcare settings. This should be taken into account when deciding whether to implement any FFP2 decontamination programs in a healthcare facility.

The experiments used a vast array of viruses (both enveloped and non-enveloped), bacteria (including some spore forming bacteria), and fungi. The methods, even when using similar technology, cannot be compared directly because there were differences in protocols (tested pathogens; applied temperature, ppm, concentration, humidity) and study designs.

Sterilization was reached at least some of the time when using ethylene oxide, gaseous hydrogen peroxide, either alone or in combination with peracetic acid, moist heat, ozone, peracetic acid (dry fogging system), UVGI, either alone or in combination with humidity, heat, and steam. Only the two studies using gaseous hydrogen peroxide combined with peracetic acid were able to sterilize all of the tested microorganisms in all experiments. Moist heat and UVGI were often tested, but only rarely resulted in sterilization. A 3log reduction as an endpoint was reached by most of the tested methods except for non-antimicrobial wipes. There, however, a number of methods that failed fit and filtration testing.

Masks reprocessed by ethylene oxide, gaseous hydrogen peroxide, and peracetic acid dry fogging all passed fit testing and filtration capacity. Microwave generated moist heat also had little impact on mask integrity, fit testing and filtration capacity, most of the time. While ozone performed very well for the facepieces, there was a tendency to degrade elastic bands, which could pose a risk of failure during a risky procedure in daily practice. Saturated steam in autoclaves was promising in some studies but failed in others. Most likely, this is due to the overall quality of masks and manufacturing. Though we included all data on mask integrity, fitting and filtration capacity in a “pass/fail” manner, it is important to note that applied testing was very heterogeneous, ranging from simple visual inspection to very complex experiments.

Few of the studies looked at bioaccumulation of physical soil in the masks from breathing. When analyzing decontamination methods, most papers didn’t take into account the bioaccumulation of protein from human breath, which could conceivably be significant, especially when performing numerous decontamination cycles. One study found the protein in exhaled breath condensate to be around 1.02 μg/ml [40]. One of the studies in this review looked at this data more closely, and found that the protein from human breath condensate accumulates at ~ 0.34 μg/minute breathing time [2]. Considering that physical soil affects the efficacy of decontamination, and that a soiled surface needs to be cleaned before it can be disinfected or sterilized, this area needs further study.

COVID-19 is a disproportionate threat to low income and low resource environments, where access to care, supplies or testing is challenging, and the living environment does not allow social distancing. Because many of the decontamination methods require costly equipment and specific infrastructure, special consideration should be given to studying simple, lower cost solutions. Some of the identified methodologies using a rice cooker, boiling pot of water, oven, or steam bag in a microwave, are potential procedures to be used in lower-resource settings or at home.

As single-use N95/FFP2 respirators do not tolerate washing with ordinary detergent, they may not be free of soiling. This interferes with the principle of disinfection and sterilization, that such procedures only can be effective if medical equipment has passed through a validated cleaning process. Thus, collecting, reprocessing and redistributing must be organized individually, which engenders a host of logistical challenges. Mask storage before reprocessing may also be a challenge, to avoid growing molds. Methods and logistical issues have a cost, which is much higher than the price per new mask. Furthermore, the capacity of reprocessing equipment such as plasma sterilizers or UVC-lamps is often limited and thus, not applicable in larger healthcare settings where thousands of N95/FFP2 respirators are needed every day.

## Limitations

The majority of the identified studies were from the grey literature, were preprints, or were not yet peer-reviewed. Many studies were conducted by industry, and may have also be subject to reporting bias. Thus, even if the quality of the experiments may have been acceptable for the most part, the generalization of the efficacy of tested methods is limited. Still, we deemed the extraordinary situation of the current COVID-19 pandemic as a reason to justify the inclusion of as many methods for mask reprocessing as possible, and to encourage further testing of the efficacy of some of the methods.

## Conclusion

In conclusion, as a principle, no disposable N95/FFP2 respirators should be reused. Reprocessing methods offer limited safety, particularly when protocols are not individualized. Mask decontamination and reuse is more expensive than purchasing new masks, and thus, can only be justified in situations of interrupted supply, and not for economic reasons. Finally, the selection of disinfection or sterilization methods for N95/FFP2 respirators must take into account the availability of effective decontamination methods, the achievable local turnover capacity as well as the individual characteristics of the masks, as they vary by manufacturer. Future research should take into consideration the biological buildup of exhaled breath, the individual characteristics of masks, and the testing of reprocessed masks in clinical conditions.

## Supplementary Information

**Additional file 1**. Search strategy for the review.

## Data Availability

All of our data is publicly available, that which is not has been sent to ARIC in the form of a Additional file 1.
