# Supplementary material for: Decontaminating N95/FFP2 masks for reuse during the COVID-19 epidemic: a systematic review
Source: Antimicrob Resist Infect Control. 2021 Oct 11;10:144. doi: 10.1186/s13756-021-00993-w (PMC8503730; doi:10.1186/s13756-021-00993-w)
Supplement: Supplementary file 1 — Additional file 1. Search strategy for the review. [file 13756_2021_993_MOESM1_ESM.pdf]

(N95 OR FFP2 OR KN95) AND (decontamination OR disinfection OR sterilization) AND (reuse OR reprocessing OR reusing) AND (coronavirus OR “COVID 19” OR "SARS CoV-2" OR stearothermophilus OR influenza)
